# 3-Methylxanthine production through biodegradation of theobromine by *Aspergillus sydowii* PT-2

**DOI:** 10.1186/s12866-020-01951-z

**Published:** 2020-08-27

**Authors:** Binxing Zhou, Cunqiang Ma, Chengqin Zheng, Tao Xia, Bingsong Ma, Xiaohui Liu

**Affiliations:** 1 College of Longrun Pu-erh Tea, Yunnan Agricultural University, Kunming, 650201 Yunnan China; 2 Henan Key Laboratory of Tea Comprehensive Utilization in South Henan, Xinyang Agriculture and Forestry University, Xinyang, 464000 Henan China; 3 Kunming Dapu Tea Industry Co., Ltd, Kunming, 650224 Yunnan China; 4 State Key Laboratory of Tea Plant Biology and Utilization, Anhui Agricultural University, Hefei, 230036 Anhui China

**Keywords:** *Aspergillus*, Tea, Bioconversion, Theobromine, 3-Methylxanthine

## Abstract

**Background:**

Methylxanthines, including caffeine, theobromine and theophylline, are natural and synthetic compounds in tea, which could be metabolized by certain kinds of bacteria and fungi. Previous studies confirmed that several microbial isolates from Pu-erh tea could degrade and convert caffeine and theophylline. We speculated that these candidate isolates also could degrade and convert theobromine through N-demethylation and oxidation. In this study, seven tea-derived fungal strains were inoculated into various theobromine agar medias and theobromine liquid mediums to assess their capacity in theobromine utilization. Related metabolites with theobromine degradation were detected by using HPLC in the liquid culture to investigate their potential application in the production of 3-methylxanthine.

**Results:**

Based on theobromine utilization capacity, *Aspergillus niger* PT-1, *Aspergillus sydowii* PT-2, *Aspergillus ustus* PT-6 and *Aspergillus tamarii* PT-7 have demonstrated the potential for theobromine biodegradation. Particularly, *A. sydowii* PT-2 and *A. tamarii* PT-7 could degrade theobromine significantly (*p* < 0.05) in all given liquid mediums. 3,7-Dimethyluric acid, 3-methylxanthine, 7-methylxanthine, 3-methyluric acid, xanthine, and uric acid were detected in *A. sydowii* PT-2 and *A. tamarii* PT-7 culture, respectively, which confirmed the existence of N-demethylation and oxidation in theobromine catabolism. 3-Methylxanthine was common and main demethylated metabolite of theobromine in the liquid culture. 3-Methylxanthine in *A. sydowii* PT-2 culture showed a linear relation with initial theobromine concentrations that 177.12 ± 14.06 mg/L 3-methylxanthine was accumulated in TLM-S with 300 mg/L theobromine. Additionally, pH at 5 and metal ion of Fe^2+^ promoted 3-methylxanthine production significantly (*p* < 0.05).

**Conclusions:**

This study is the first to confirm that *A. sydowii* PT-2 and *A. tamarii* PT-7 degrade theobromine through N-demethylation and oxidation, respectively. *A. sydowii* PT-2 showed the potential application in 3-methylxanthine production with theobromine as feedstock through the N-demethylation at N-7 position.

## Background

Methylxanthines are natural and synthetic compounds found in many foods, drinks, pharmaceuticals, and cosmetics [1]. Caffeine (1,3,7-trimethylxanthine), theobromine (3,7-dimethylxanthine) and theophylline (1,3-dimethylxanthine) are most popular and well-known methylxanthines in tea [2]. Both theobromine and theophylline have a close connection with caffeine metabolism in the physiology of tea plant (*Camellia sinensis*), and the former is precursor of caffeine biosynthesis and the latter is a transient metabolite of caffeine biodegradation [3, 4]. Caffeine level remains stable in the processing of general teas (green tea, black tea, oolong tea and white tea) [5, 6]. However, the participation of various microorganisms induced the change of caffeine content in the processing of Pu-erh tea and other dark teas [7, 8].

Pu-erh tea is a Chinese dark tea produced mainly in Yunan province [9]. Its unique taste and aroma is achieved by natural microorganisms involved in solid-state fermentation [10]. Microorganisms, including bacteria and fungi, have profound impact on substance metabolisms and contributed to the quality formation of Pu-erh tea [11–13]. *Aspergillus niger*, *Aspergillus tubingensis*, *Aspergillus fumigatus*, *Aspergillus luchuensis*, *Aspergillus awamor*i, *Aspergillus tamarii*, *Blastobotrys adeninivorans*, *Candida tropicalis*, *Fusarium graminearum*, *Pichia farinosa*, *Rasamsonia byssochlamydoides*, *Rasamsonia emersonii*, *Rasamsonia cylindrospora*, *Rhizomucor pusillus*, *Rhizomucor tauricus* and *Thermomyces lanuginosus* were detected consecutively in Pu-erh tea [14–17]. Among seven microbial isolates from Pu-erh tea, *Aspergillus sydowii* have been confirmed to convert degraded caffeine to theophylline, *Aspergillus ustus* and *A. tamarii* showed theophylline degradation capacity in liquid culture, respectively [18–20].

Methylxanthines are extensively metabolized in the liver by the cytochrome P450 (CYP450) oxidase enzyme system, mainly through related N-demethylation and oxidation [21]. Although caffeine and other methylxanthines are toxic to most bacteria and invertebrates [22], several bacteria and fungi, including *Pseudomonas* sp. [23, 24], *Pseudomonas putida* [25, 26], *Serratia marcescens, Fusarium solani* [27, 28], *Stemphyllium* sp., *A. tamarii* and *Penicillium commune* [29], have evolved the ability to metabolize caffeine. Two possible pathways of caffeine catabolism, such as N-demethylation and oxidation, are found in microorganisms, which are similar to that in animals and humans [30]. More than one N-demethylases and oxidases, such as caffeine oxidase, xanthine oxidase and theobromine oxidase, participate into the N-demethylation and oxidation [31–33]. Genes (*ndmA*, *ndmB*, *ndmC*, *ndmD* and *ndmE*) isolated from *P. putida* are responsible for the entire demethylation pathway [34, 35]. Additionally, genes *cdhABC* and *tmuDHM* identified in *Pseudomonas* sp. strain CBB1 are associated with the oxidation of caffeine and trimethyluric acid, respectively [36, 37]. Therefore, we speculated that those seven microbial isolates from Pu-erh tea also could degrade and convert theobromine through N-demethylation and oxidation.

As the second most common methylxanthine in tea, theobromine dilates blood vessels, especially coronary arteries, lowers blood pressure and increases heart rate [38]. Until now, a bacterial strain *P. putida* isolated from tea garden soil was demonstrated to degrade theobromine [26]. Additionally, *A. niger*, *Talaromyces marneffei* and *Talaromyces verruculosus* isolated from cocoa pod husks were demonstrated to degrade theobromine [39]. In this work, seven tea-derived fungal strains isolated from Pu-erh tea were used to investigate their capacity and characterization in theobromine degradation. It is confirmed that *Aspergillus sydowii* PT-2 and *Aspergillus tamarii* PT-7 could degrade theobromine in the liquid culture. Analysis of theobromine degradation metabolites and pathways revealed that 3-methylxanthine was main degradation product of theobromine in *A. sydowii* PT-2 culture through the N-demethylation at N-7 position. The results showed the application of *A. sydowii* PT-2 in the production of 3-methylxanthine with theobromine as feedstock.

## Results

### Evaluation results of tea-derived fungi in theobromine utilization

**Fig. 1:**
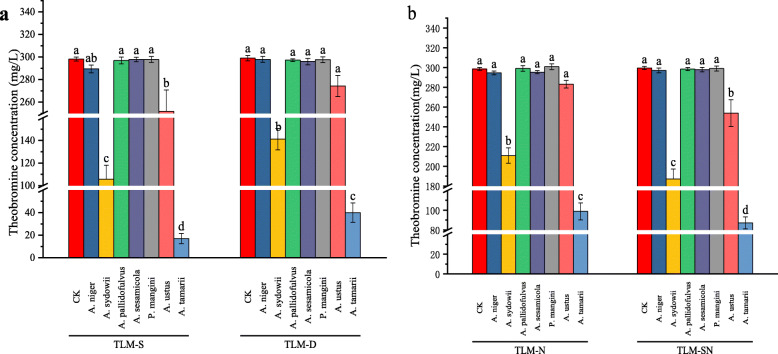
Theobromine degradation capacity of tea-derived fungi in the liquid culture. **a** TLM-S = Theobromine liquid medium with sucrose as carbon source; TLM-D = Theobromine liquid medium with dextrose with sucrose as carbon source; **b** TLM-N = Theobromine liquid medium with ammonium sulphate as nitrogen source; TLM-SN = Theobromine liquid medium with sucrose and ammonium sulphate as carbon and nitrogen sources, respectively. Biocidal treatment without inoculation was defined as the control. All data were present by mean value ± SD of three biological replications. The lowercase letters indicated a significant difference at *p* < 0.05 levels by using Tukey’s multiple comparison test for one-way ANOVA. The different letters show significant differences

To assess theobromine utilization capacity of tea-derived fungi, each microbial isolate was inoculated into different theobromine agar medias (TAM) and theobromine liquid mediums (TLM), respectively. Colony diameters and theobromine concentrations were determined after cultivation at 30 °C for 5 days. Colony diameters and sporulation time on TAM are recorded in Table 1, and theobromine concentrations in TLM are presented in Fig. 1. As shown in Table 1, apart from *Aspergillus pallidofulvus* PT-3 and *Penicillium mangini* PT-5, other microbial isolates had relatively high theobromine utilization capacity, such as *Aspergillus niger* PT-1, *A. sydowii* PT-2, *Aspergillus sesamicola* PT-4, *Aspergillus ustus* PT-6 and *A. tamarii* PT-7. Comparison of colony diameters on different TAM showed that dextrose or sucrose as carbon source could promote theobromine utilization partly. TAM-S with the maximal colony diameter was most suitable for theobromine utilization by candidate fungal strains.

**Table 1:** Colony diameter and sporulation time of tea-derived fungi on theobromine agar medias

Tea-derived fungi	Colony diameter (cm)				Total diameter (cm)	Day of sporulation			
	TAM-D	TAM-N	TAM-S	TAM-T		TAM-D	TAM-N	TAM-S	TAM-T
*A. niger* PT-1	3.2 ± 0.1	2.9 ± 0.1	3.5 ± 0.1	1.0 ± 0.1	10.6 ± 0.3	4	–	5	–
*A. sydowii* PT-2	4.0 ± 0.2	3.3 ± 0.1	4.5 ± 0.1	1.8 ± 0.1	13.7 ± 0.4	5	–	4	–
*A. pallidofulvus* PT-3	2.2 ± 0.2	1.2 ± 0.2	2.6 ± 0.1	0	6.0 ± 0.4	–	–	–	–
*A. sesamicola* PT-4	2.2 ± 0.1	2.1 ± 0.5	2.4 ± 0.2	0.5 ± 0.1	7.2 ± 0.2	5	–	4	–
*P. mangini* PT-5	1.6 ± 0.1	1.5 ± 0.2	2.1 ± 0.1	0	5.2 ± 0.4	–	–	–	–
*A. ustus* PT-6	3.5 ± 0.1	2.7 ± 0.1	4.0 ± 0.2	1.8 ± 0.1	12.0 ± 0.2	4	5	4	–
*A. tamarii* PT-7	5.6 ± 0.2	4.6 ± 0.2	6.0 ± 0.2	3.0 ± 0.3	19.1 ± 0.4	3	4	2	4

*TAM-D* theobromine agar media with dextrose as carbon source, *TAM-N* theobromine agar media with ammonium sulphate as nitrogen source, *TAM-S* theobromine agar media with sucrose as carbon source, *TAM-T* theobromine agar media with theobromine as sole carbon and nitrogen source

In this study, TLM-S, TLM-D, TLM-N, TLM-SN were prepared to select potential theobromine-degrading fungi and optimal medium in the liquid culture. As shown in Fig. 1, due to the difference in cultivation modes, *A. pallidofulvus* PT-3, *A. sesamicola* PT-4 and *P. mangini* PT-5 could not utilize theobromine completely in all given TLM. *A. niger* PT-1 just used the theobromine in TLM-S slightly. Only *A. sydowii* PT-2, *A. ustus* PT-6 and *A. tamarii* PT-7 could utilize theobromine largely in the liquid culture. The additional carbon source promoted theobromine utilization capacity of *A. sydowii* PT-2 and *A. tamarii* PT-7 through enhancement of cell density in the liquid culture [19]. Particularly, the highest theobromine removal rate was found in TLM-S for the potential theobromine-degrading fungi, including *A. niger* PT-1, *A. sydowii* PT-2, *A. ustus* PT-6 and *A. tamarii* PT-7*.* The composition of TLM-S was therefore chosen as the optimal medium to investigate theobromine degradation metabolites in the liquid culture.

### Theobromine degradation characterization in liquid culture

**Fig. 2:**
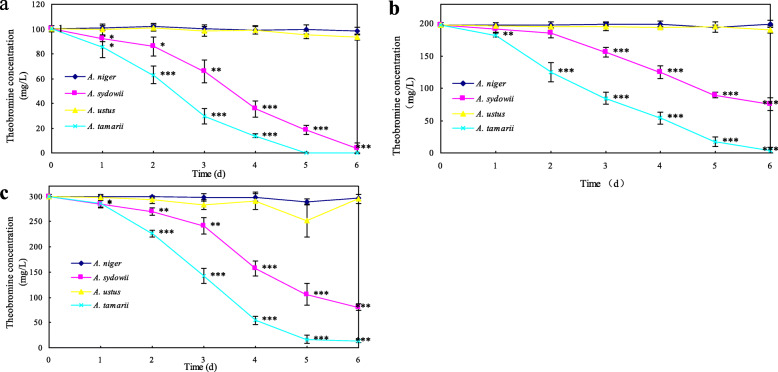
Effect of candidate isolates on theobromine degradation in different substrate concentrations. Theobromine concentrations were 100 mg/L (**a**), 200 mg/L (**b**), and 300 mg/L (**c**), respectively. All data were present by mean value ± SD of three replications. The significance level between *A. niger* PT-1 and other candidate isolates was assessed by using the independent t-test of SPSS 20.0. * *p* < 0.05; ** *p* < 0.01; *** *p* < 0.001

*A.niger* PT-1, *A. sydowii* PT-2, *A. ustus* PT-6 and *A. tamarii* PT-7 were inoculated into TLM-S with an increasing theobromine concentration (100, 200 and 300 mg/L, respectively), and Tissue-culture bottles were incubated in an orbital shaker (130 rpm, 30 °C), respectively. The inoculated bottles were took every 24 h for the determination of theobromine and related metabolites by using high-performance liquid chromatography (HPLC). Theobromine concentrations (Additional file 1: Table S1) are presented in Fig. 2. Significant difference (*p* < 0.05) was found in theobromine concentrations between four candidate isolates. Theobromine decreased slightly (*p* > 0.05) in all concentrations inoculated by *A. niger* PT-1 and *A. ustus* PT-6, which showed a limited theobromine utilization capacity in the liquid culture. In time-course experiments over a period of 6 days, *A. tamarii* PT-7 could degrade almost all the theobromine in the liquid culture. However, theobromine degradation capacity of *A. sydowii* PT-2 was limited with theobromine removal rates about 61.92 and 73.12% in high substrate concentrations of 200 mg/L and 300 mg/L, respectively.

Theobromine catabolic intermediates were identified by HPLC using internal standard method (Table 2). 3,7-Dimethyluric acid, 3-methylxanthine, 7-methylxanthine, 3-methyluric acid, xanthine and uric acid were detected consecutively in the liquid culture. The detected metabolites showed that both N-demethylation and oxidation were found in theobromine catabolism. Quantitative analysis indicated that 3-methylxanthine was common and main demethylated metabolite through N-demethylation at the N-7 position of theobromine in *A. sydowii* PT-2 and *A. tamarii* PT-7 culture*.* 7-Methylxanthine was inferred as the demethylated product through the N-3 demethylation in *A. tamarii* PT-7 culture. Xanthine was a further demethylated metabolite found in *A. tamarii* PT-7 culture through N-3 demethylation of 3-methylxanthine or N-7 demethylation of 7-methylxanthine. In *A. ustus* PT-6 and *A. tamarii* PT-7 culture, 3,7-dimethyluric acid, 3-methyluric acid and uric acid were direct oxidation products from theobromine, 3-methylxanthine and xanthine, respectively.

**Table 2:** Theobromine degradation metabolites detected in the liquid culture of four candidate isolates

Candidate isolates	Metabolites
*A. niger* PT-1	Not found
*A. sydowii* PT-2	3-Methylxanthine and xanthine
*A. ustus* PT-6	3,7-Dimethyluric acid and 3-methyluric acid
*A. tamarii* PT-7	3,7-Dimethyluric acid, 3-methylxanthine, 7-methylxanthine, 3-methyluric acid, xanthine and uric acid

TLM-S inoculated by candidate isolates were analyzed by HPLC for 3,7-dimethyluric acid, 3-methylxanthine, 7-methylxanthine, 3-methyluric acid, 7-methyluric acid, xanthine and uric acid

### Production of 3-methylxanthine through theobromine biodegradation

**Fig. 3:**
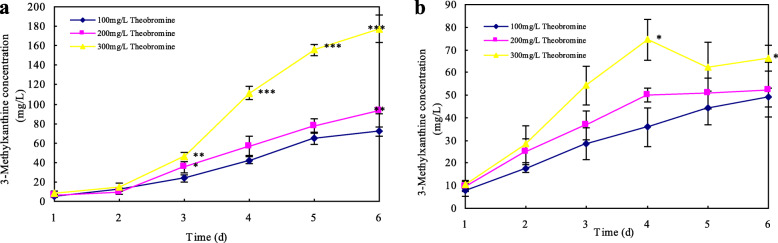
Comparison of *A. sydowii* PT-2 (**a**) and *A. tamarii* PT-7 (**b**) on 3-menthylxanthine accumulation in the liquid culture. All data were present by mean value ± SD of three biological replications. The significance level was assessed by using the independent t-test of SPSS 20.0 compared with that in 100 mg/L of theobromine . * *p* < 0.05; ** *p* < 0.01; *** *p* < 0.001

3-Methylxanthine and other methylxanthines have been shown various biomedical effects as adenosine receptors and inhibitors of Primary Amine Oxidase [39, 40]. Due to high accumulation of 3-methylxanthine, 3-methylxanthine concentrations were determined by HPLC in *A. sydowii* PT-2 and *A. tamarii* PT-7 culture, respectively. 3-Methylxanthine concentrations are recorded in Additional file 1: Table S2 and presented in Fig. 3. The accumulation of 3-methylxanthine increased along with theobromine degradation since it was detected in the liquid culture after cultivation for 24 h. Over a 6-day period cultivation of *A. sydowii* PT-2 (Fig. 3a), 71.84 ± 4.44 mg/L, 92.81 ± 2.86 mg/L and 177.12 ± 14.06 mg/L of 3-methylxanthine were produced and increased significantly (*p* < 0.05) with an increasing initial theobromine concentration, respectively, showing a linear relationship between theobromine degradation and 3-methylxanthine accumulation. However, the accumulation of 3-methylxanthine maintained at a low level about 66.31 ± 5.68 mg/L in *A. tamarii* PT-7 culture with 300 mg/L theobromine, which was far below that in *A. sydowii* PT-2 culture. Generally, *A. sydowii* PT-2 showed its advantage in the production of 3-methylxanthine with 300 mg/L theobromine as feedstock.

**Fig. 4:**
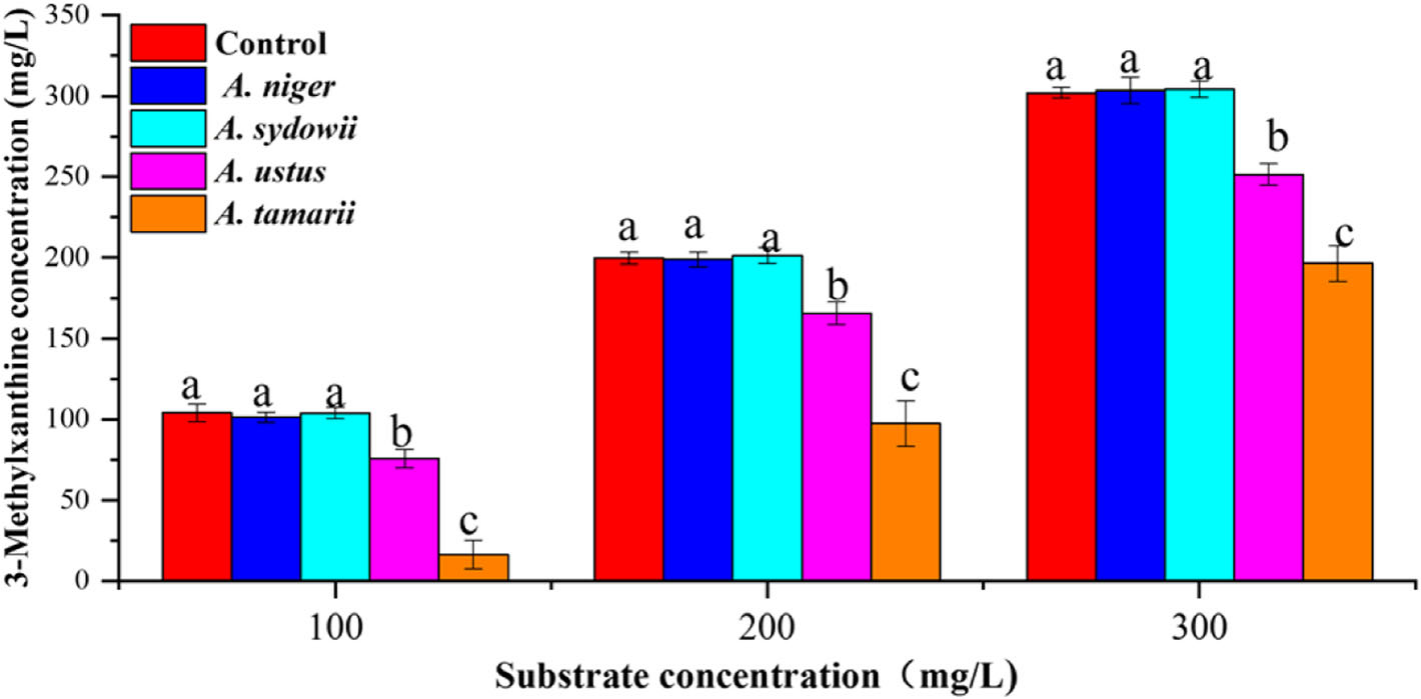
Effect of candidate isolates on 3-methylxanthine metabolism. Biocidal treatment without inoculation was defined as the control. All data were present by mean value ± SD of three biological replications. The lowercase letters indicated a significant difference at *p* < 0.05 level by using one-way ANOVA of SPSS 20.0 between different candidate isolates at same substrate concentration. The different letters show significant differences

The non-linear relationship between theorbromine degradation and 3-methylxanthine accumulation in *A. tamarii* PT-7 culture indicated that as the main intermediate of theobromine degradation, 3-methylxanthine might be degraded by *A. tamarii* PT-7 and other candidate isolates in the liquid culture. To investigate 3-mehylxanthine metabolism, four candidate isolates were inoculated into a linearly increasing concentration of 3-methylxanthine from 100 mg/L to 300 mg/L, 3-methylxanthine and related metabolites were determined by HPLC (Fig. 4). Compared with other isolates, *A. sydowii* PT-2 and *A. tamarii* PT-7 could reduce 3-methylxanthine significantly (*p* < 0.05) in all given concentrations. Particularly, *A. tamarii* PT-7 degrade almost all 3-methylxanthine in a low substrate concentration (100 mg/L 3-methylxanthine), and maintained a relatively high removal rate about 34.97% in 300 mg/L substrate concentration after cultivation for 5 days. Through the analysis of related metabolites with 3-methylxanthine degradation (Additional file 1: Table S3), 3-methyluric acid, xanthine and uric acid were detected in the liquid culture, respectively. Associated with the metabolites detected in theobromine degradation, xanthine was demethylated product from 3-methylxanthine through N-3 demethylation. Alternatively, 3-methyluric acid and uric acid were direct oxidative products from 3-methylxanthine and xanthine at the C-8 position, respectively.

### Effects of pH and metal ions on 3-methylxanthine production

**Fig. 5:**
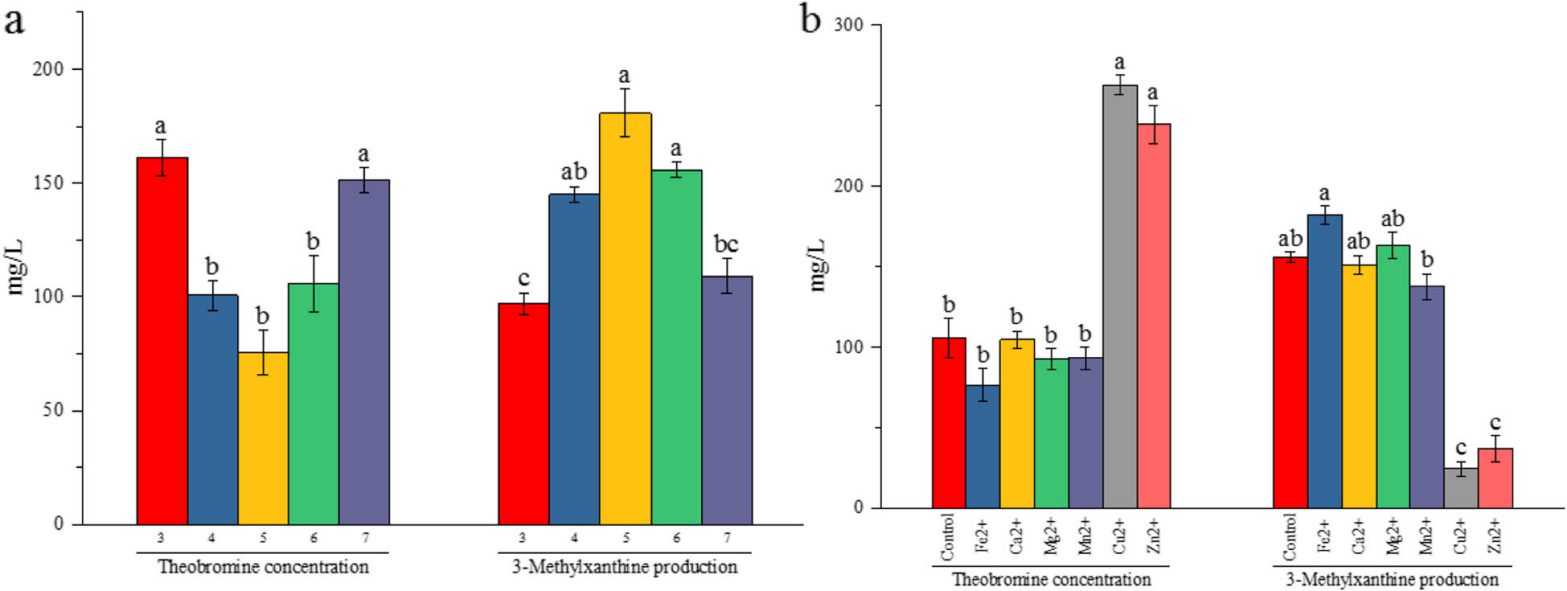
Effects of pH (**a**) and metal ions (**b**) on theobromine degradation and 3-methylxanthine production in *A. sydowii* PT-2 culture, respectively. The pH in the inoculated mediums was adjusted by phosphate buffer. Metal ions were added into the culture solution at a concentration of 2 mM. The culture solution without extra addition was defined as the control. The reaction (pH 6.0) was carried out at 30 °C for 5 days on an incubator shaker (130 rpm). Data are presented as mean value ± SD of three biological replications. The lowercase letters indicated a significant difference at *p* < 0.05 level by using Tukey’s multiple comparison test for one-way ANOVA. The different letters show significant differences

The lower degradation capacity of 3-methylxanthine in liquid culture (Fig. 4) confirmed that *A. sydowii* PT-2 had application potential in production of 3-methylxanthine with theobromine as feedstock. Metal ions and pH were principal factors influencing theobromine biodegradation and 3-methylxanthine production. Two series of experiments, such as a pH range from 3 to 7 and various metal ions, including Fe^2+^, Ca^2+^, Mg^2+^, Mn^2+^, Cu^2+^ and Zn^2+^, were prepared in TLM-S to investigate the influences of pH and metal ions, respectively. *A. sydowii* PT-2 exhibited a high sensitivity to pH, showing the best theobromine degradation and 3-methylxanthine production at pH 5 (Fig. 5a). Cu^2+^ and Zn^2+^ restrained theobromine degradation and 3-methylxanthine production significantly (*p* < 0.05), only Fe^2+^ promoted 3-methylxanthine production significantly (*p* < 0.05) compared with the control (Fig. 5b).

## Discussion

Besides the traditional Traube synthesis, bioconversion offers an alternative way to produce 3-methylxanthine by using appropriate starter strain and precursor substances. Algharrawi et al. reconstructed an engineered *Escherichia coli* with genes *ndmA* and *ndmD* from *P. putida*, capable of producing 3-methylxanthine from exogenously fed theophylline [41]. Mckeague et al. engineered the eukaryotic microbial host *Saccharomyces cerevisiae* for the de novo biosynthesis of methylxanthines [42]. Additionally, 3-methylxanthine was main intermediate metabolite of theobromine through the N-7 demethylation by relevant fungi [31].

Because of dominant microbe in the solid-state fermentation causing the significant reduction of caffeine content [18], Pu-erh tea could be used to select effective strains converting theobromine to 3-methylxanthine. For seven tea-derived isolates, including *A. niger* PT-1, *A. sydowii* PT-2, *A. pallidofulvus* PT-3, *A. sesamicola* PT-4, *P. manginii* PT-5, *A. ustus* PT-6 and *A. tamarii* PT-7, five confirmed theobromine utilization capacity when cultured on TAM. The further screening in TLM showed that *A. niger* PT-1, *A. sydowii* PT-2, *A. ustus* PT-6 and *A. tamarii* PT-7 had relatively high theobromine degradation capacity in TLM-S after cultivation for 5 days at 30 °C. Particularly, *A. sydowii* PT-2 and *A. tamarii* PT-7 (Fig. 1) could degrade amount of theobromine (64.5 and 94.3%, respectively).

It was established that the isolates generally preferred TLM-S in which extra sucrose enhanced theobromine degradation efficiency. Theobromine degradation efficiency of four candidate isolates was entirely different (Fig. 2). Theobromine degradation capacity of *A. niger* PT-1 and *A. ustus* PT-6 were limited in the liquid culture, which might be related to cultivation method and medium components. For relatively high theobromine degradation efficiency, *A. tamarii* PT-7 and *A. sydowii* PT-2 were selected to investigate theobromine degradation pathway and application potential in the production of 3-methylxanthine.

The detected metabolites, including 3,7-dimethyluric acid, 3-methylxanthine, 7-methylxanthine, 3-methyluric acid, xanthine and uric acid, confirmed the existence of N-demethylation and oxidation in theobromine catabolism. N-demethylation happened at N-3 and N-7 positions in purine ring catalyzed by related N-demethylase, and oxidation happened at the C-8 position catalyzed by related xanthine oxidase [26, 31, 33]. For *A. sydowii* PT-2, the likely theobromine catabolic process was described as follows: theobromine→3-methylxanthine→xanthine. Except the above-mentioned process, other alternative processes also could be found in *A. tamarii* PT-7 culture. Such as the possible N-demethylation pathways were described as follows: theobromine →7-methylxanthine; 3,7-dimethyluric acid →3-methyluric acid. The possible oxidizing reaction happened in theobromine, 3-methylxanthine and xanthine as precursor substance as follows: theobromine→ 3,7-dimethyluric acid; 3-methylxanthine→3-methyluric acid and xanthine →uric acid. We speculated that the related N-demethylase and xanthine oxidase released by *A. sydowii* PT-2 and *A. tamarii* PT-7 caused the difference in theobromine catabolism. Although caffeine catabolic pathways in tea plant and microorganisms are relatively clear [3, 4, 25–27], theobromine catabolic pathways in the liquid culture of potential microorganisms have not been defined, which deserves further research.

We confirmed that *A. sydowii* mainly produced theophylline through N-demethylation at the N-7 position of caffeine, other N-demethylated metabolites, such as 1,7-dimethylxanthine, 7-methylxanthine and 3-methylxanthine, were detected during tea fermentation, which showed that *A. sydowii* could release related N-demethylase [43]. In this study, apart from caffeine, *A. sydowii* PT-2 also could remove the N-7 methyl of theobromine to formulate 3-methylxanthine. Although *A. ustus* largely converted theophylline into 3-methylxanthine through the N-1 demethylation [19], absence of N-demethylase removing the N-7 methyl limited theobromine degradation efficiency in the liquid culture. *A. tamarii* PT-7 exhibited broad-spectrum capacity in methylxanthines degradation, including theobromine, theophylline and 3-methylxanthine by releasing various N-demethylases and oxidases, respectively. The high degradation of 3-methylxanthine reduced the accumulation of 3-methylxanthine in *A. tamarii* PT-7 culture. Therefore, *A. sydowii* PT-2 was best in production of 3-methylxanthine with theobromine as feedstock.

Substrate concentration, pH and metal ions had profound impacts on theobromine degradation and 3-methylxanthine production. *A. sydowii* PT-2 produced the maximum accumulation of 3-methylxanthine in the liquid culture of 300 mg/L theobromine. Comparisons showed that the optimal pH was 5 and Fe^2+^ promoted the conversion of theobromine into 3-methylxanthine significantly (*p* < 0.05), which provided optimum condition for the growth of *A. sydowii* PT-2 and enzymatic reaction of relevant N-demethylase.

## Conclusions

This paper describes related metabolites with theobromine degradation and explores potential application of tea-derived fungi in the production of 3-methylxanthine. Among seven microbial isolates from Pu-erh tea, both *A. sydowii* PT-2 and *A. tamarii* PT-7 showed higher theobromine degradation capacity in TAM and TLM. 3,7-Dimethyluric acid, 3-methylxanthine, 7-methylxanthine, 3-methyluric acid, xanthine and uric acid were detected by using HPLC in *A. sydowii* PT-2 and *A. tamarii* PT-7 culture, respectively, which confirmed the existence of N-demethylation and oxidation in theobromine catabolism. Compared with that in *A. tamarii* PT-7 culture, 3-methylxanthine was accumulated largely in *A. sydowii* PT-2 culture along with theobromine degradation and showed a linear relation with initial theobromine concentration. *A. sydowii* PT-2 was an appropriate starter strain most suitable for the production of 3-methylxanthine, which could produce 177.12 ± 14.06 mg/L 3-methylxanthine in TLM-S with 300 mg/L theobromine. Additionally, pH at 5 and metal ion of Fe^2+^ promoted the production of 3-methylxanthine significantly (*p* < 0.05). This paper presents an alternative way for 3-methylxanthine production through the microbial conversion of *A. sydowii* PT-2 with theobromine as feedstock.

## Methods

### Strains and reagents

Tea-derived fungal strains (Table 3) used in this study were isolated from Pu-erh tea and identified based on colony characteristics, conidial structure and PCR amplified sequences, and stored at − 20 °C in our microbiology laboratory before further processing. Theobromine, 3,7-dimethyluric acid, 3-methylxanthine, 7-methylxanthine, 3-methyluric acid, 7-methyluric acid, xanthine and uric acid were purchased from Sigma-Aldrich Co., Ltd. HPLC-grade acetonitrile and ammonium formate were purchased from Thermo Fisher Scientific Co., Ltd. Other reagents, including agar, dextrose, sucrose and ammonium sulphate, were analytical grade.

**Table 3:** Strains information of tea-derived fungi used in this work

Isolate ^a^	Primers	Fragments (bp)	Accession No. ^b^	Species	Strain No.	Homology
PT-1	ITS1/ITS4	546	MT065763	*Aspergillus niger*	NCBT 110A	99.8%
PT-2	ITS1/ITS4	516	MT065764	*Aspergillus sydowii*	NRRL 250	99.8%
PT-3	ITS1/ITS4	541	MT065765	*Aspergillus pallidofulvus*	NRRL 4789	99.9%
	Bt2a/Bt2b	516	MT084116			
	CF1L/CF4	765	MT084120			
PT-4	ITS1/ITS4	532	MT065766	*Aspergillus sesamicola*	CBS 137324	99.8%
	Bt2a/Bt2b	515	MT084117			
	CF1L/CF4	757	MT084121			
PT-5	ITS1/ITS4	525	MT065767	*Penicillium manginii*	CBS 253.31	99.6%
	Bt2a/Bt2b	420	MT084118			
PT-6	ITS1/ITS4	502	MT065768	*Aspergillus ustus*	NRRL 275	100%
	CF1L/CF4	694	MT084122			
PT-7	ITS1/ITS4	532	MT065769	*Aspergillus tamarii*	NRRL 20818	99.9%
	Bt2a/Bt2b	476	MT084119			
	CF1L/CF4	715	MT084123			

^a^ Those strains were stored in the microbiology laboratory of Yunnan Agricultural University with the number from PT-1 to PT-7, which can be accessed for reproducibility if need

^b^ GenBank/EMBL/DDBJ accession number

### Evaluation of growth of tea-derived fungi on theobromine agar medias

For each strain, spore suspension was adjusted to 1.0 × 10^7^ CFU/mL for inoculation after cultivation on PDA media at 30 °C for 72 h, respectively [19]. Four kinds of TAM contained 20 g/L agar and 600 mg/L theobromine were carried out to evaluate theobromine utilization, which included theobromine agar media with 2.0 g/L dextrose as carbon source (TAM-D), theobromine agar media with 1.01 g/L ammonium sulphate as nitrogen source (TAM-N), theobromine agar media with 2.0 g/L sucrose as carbon source (TAM-S) and theobromine agar media only with theobromine as sole carbon and nitrogen source (TAM-T), respectively. Plates of each TAM were inoculated with 10 uL spore suspension and incubated at 30 °C. At 24-h intervals for 5 days, colony diameters were measured [44]. The isolated strains were categorized based on their total colony diameters as follows: low theobromine utilization = diameter ≤ 7.9 cm; moderate theobromine utilization = diameter 8.0-15.9 cm; high theobromine utilization = diameter ≥ 16.0 cm. Tea-derived fungi selected for further study were those that showed at least moderate theobromine utilization (diameter ≥ 8.0 cm) on agar medias.

### Assessment of theobromine-degrading fungi in theobromine liquid mediums

Theobromine liquid medium (TLM) was prepared by using 4.0 g/L NaNO_3_, 1.3 g/L KH_2_PO_4_, 0.19 g/L Na_2_HPO_4_·7H_2_O, 0.26 g/L CaCl_2_·2H_2_O, 0.15 g/L MgSO_4_, 2.0 g/L sucrose and 300 mg/L theobromine in distilled water [45]. To investigate the influence of carbon and nitrogen source on theobromine degradation, the modifications used either 5 g/L sucrose or 10 g/L dextrose as carbon source in theobromine liquid medium with sucrose as carbon source (TLM-S) or theobromine liquid medium with dextrose as carbon source (TLM-D), and 1.01 g/L ammonium sulphate as nitrogen source in theobromine liquid medium with ammonium sulphate as nitrogen source (TLM-N), and 5 g/L sucrose and 1.01 g/L ammonium sulphate in theobromine liquid medium with sucrose and ammonium sulphate as carbon and nitrogen sources (TLM-SN), respectively. The spore suspension was adjusted to 1.0 × 10^7^ CFU/mL for inoculation after eluting by using sterile saline solution with identical theobromine concentration. Both spore suspension and TLM were adjusted for pH 6.0 by phosphate buffer. For each isolate, control and experimental mediums (25 mL each) were inoculated with spore suspension with 4% inoculum size (v/v) that 1 mL spore suspension was inoculated into each medium, and biocidal treatment was defined as the control. Theobromine concentration was determined after cultivation at 30 °C for 5 days on an incubator shaker (130 rpm), respectively.

### Analysis of theobromine degradation metabolites in liquid culture

Through comparisons (Fig. 1), TLM-S therefore was chosen as the optimal medium to analyze theobromine degradation in the liquid culture. A series of TLM-S with different initial theobromine concentrations (100, 200 and 300 mg/L, respectively) were set up and a 6-day period cultivation of each selected isolate were carried out on an incubator shaker (130 rpm, 30 °C). At intervals of up to 24 h for 6 days, an aliquot of each culture was filtered through a 0.45 um syringe filter. Theobromine concentration and related metabolites were determined by HPLC using Agilent C18 chromatogram column (250 mm × 4.6 mm, 5 μm) with solvent A (100% acetonitrile) and solvent B (0.1% ammonium formate) as mobile phase [19, 31].

Standard calibration curves were prepared from solutions of theobromine, 3-methylxanthine, 7-methylxanthine, xanthine, 3,7-dimethyluric acid, 3-methyluric acid, 7-methyluric acid and uric acid. Internal standard method was used to aid in the identification of metabolites related to theobromine catabolism [19]. 3-Methylxanthine was quantificationally analyzed as the main intermediate metabolite in the liquid culture of *A. sydowii* PT-2 and *A. tamarii* PT-7, respectively.

### Influence of potential isolates on 3-methylxanthine metabolism

3-Methylxanthine liquid mediums were prepared as above described with 5 g/L sucrose as carbon source and a linearly increasing concentration of 3-methylxanthine from 100 mg/L to 300 mg/L to explore the effect of four candidate isolates, including *A. niger* PT-1, *A. sydowii* PT-2, *A. ustus* PT-6 and *A. tamarii* PT-7, respectively. Each candidate isolate was inoculated with 4% inoculum size (v/v) and 3-methylxanthine concentration was determined by HPLC after cultivation at 30 °C for 5 days on an incubator shaker (130 rpm), respectively.

### Effects of pH and metal ions on theobromine degradation and 3-methylxanthine production

Effect of pH on theobromine degradation and 3-methylxanthine production was investigated in TLM-S with a pH range from 3 to 7 adjusted by phosphate buffer [18, 46]. In order to study the effect of metal ions, Fe^2+^, Ca^2+^, Mg^2+^, Mn^2+^, Cu^2+^ and Zn^2+^ were added into the culture solution in the form of salts (viz. FeSO_4_·7H_2_O, CaCl_2_·2H_2_O, MgSO_4_, MnSO_4_·H_2_O, CuSO_4_·5H_2_O, ZnSO_4_·7H_2_O) at a concentration of 2 mM and the culture solution without extra metal ions was defined as the control [32]. Theobromine and 3-methylxanthine concentrations were determined after cultivation at 30 °C for 5 days on an incubator shaker (130 rpm), respectively.

### Statistical analysis

Three biological replications were carried out to ensure validity and repeatability. All data are presented as mean value ± standard deviation (SD). The independent t-test and Tukey’s multiple comparison tests for one-way analysis of variance (ANOVA) were carried out by using SPSS 20.0 for Windows to determine significant difference level.

## Supplementary information

**Additional file 1: Table S1.** Comparison of theobromine concentrations detected by HPLC in liquid culture of different candidate isolates. **Table S2.** Production of 3-methylxanthine in TLM-S with different substrate concentrations inoculated by *A. sydowii* PT-2 and *A. tamarii* PT-7, respectively. **Table S3.** Related metabolites with 3-methylxanthine degradation detected in the liquid culture of different candidate isolates.

## Data Availability

The data that support the findings of this study are available from the corresponding author upon reasonable request.

## Abbreviations

CYP450: Cytochrome P450
TAM-D: Theobromine agar media with dextrose as carbon source
TAM-N: Theobromine agar media with ammonium sulphate as nitrogen source
TAM-S: Theobromine agar media with sucrose as carbon source
TAM-T: Theobromine agar media with theobromine as sole carbon and nitrogen source
TLM: Theobromine liquid medium
TLM-S: Theobromine liquid medium with sucrose as carbon source
TLM-D: Theobromine liquid medium with dextrose as carbon source
TLM-N: Theobromine liquid medium with ammonium sulphate as nitrogen source
TLM-SN: Theobromine liquid medium with sucrose and ammonium sulphate as carbon and nitrogen sources
HPLC: High-performance liquid chromatography
PCR: Polymerase chain reaction
CFU: Colony forming units
PDA: Potato dextrose agar
SD: Standard deviation
SPSS: Statistical product and service solutions
ANOVA: Analysis of variance
