# Supplementary material for: 3-Methylxanthine production through biodegradation of theobromine by Aspergillus sydowii PT-2
Source: BMC Microbiol. 2020 Aug 27;20:269. doi: 10.1186/s12866-020-01951-z (PMC7453516; doi:10.1186/s12866-020-01951-z)
Supplement: Supplementary file 1 — Additional file 1: Table S1. Comparison of theobromine concentrations detected by HPLC in liquid culture of different candidate isolates. Table S2. Production of 3-methylxanthine in TLM-S with different substrate concentrations inoculated by A. sydowii PT-2 and A. tamarii PT-7, respectively. Table S3. Related metabolites with 3-methylxanthine degradation detected in the liquid culture of different candidate isolates. [file 12866_2020_1951_MOESM1_ESM.doc]

**Additional file 1: Table S1** Comparison of theobromine concentrations detected by HPLC in liquid culture of different candidate isolates.

| Theobromine concentration (mg/L) in the TLM-S with 100 mg/L theobromine. | | | | | | | |
| --- | --- | --- | --- | --- | --- | --- | --- |
| Isolates | 0 d | 1 d | 2 d | 3 d | 4 d | 5 d | 6 d |
| *A. niger* PT-1 | 100.6±2.5^Aa^ | 100.9±1.8^Aa^ | 101.9±3.0^Aa^ | 100.0±3.1^Aa^ | 99.3±3.5^Aa^ | 99.5±3.7^Aa^ | 99.0±3.2^Aa^ |
| *A. sydowii* PT-2 | 100.6±2.5^Aa^ | 92.4±2.6^ABab^ | 85.9±7.7^Bb^ | 65.8±9.4^Bb^ | 35.5±6.6^Bb^ | 18.7±3.7^Bb^ | 3.6±4.2^Bb^ |
| *A. ustus* PT-6 | 100.6±2.5^Aa^ | 99.7±4.5^ABa^ | 101.1±2.5^ABa^ | 98.2±4.0^Aa^ | 98.9±3.0^Aa^ | 99.4±2.9^Aa^ | 99.3±2.5^Aa^ |
| *A. tamarii* PT-7 | 100.6±2.5^Aa^ | 83.4±8.7^Bb^ | 63.0±7.0^Cc^ | 29.6±6.3^Cc^ | 13.8±1.8^Cc^ | 0.0±0.0^Cc^ | 0.0±0.0^Bb^ |
| Theobromine concentration (mg/L) in the TLM-S with 200 mg/L theobromine. | | | | | | | |
| Isolates | 0 d | 1 d | 2 d | 3 d | 4 d | 5 d | 6 d |
| *A. niger* PT-1 | 198.3±3.0^Aa^ | 198.3±3.1^Aa^ | 198.4±4.7^Aa^ | 199.3±3.1^Aa^ | 199.2±5.4^Aa^ | 194.3±8.1^Aa^ | 199.5±5.5^Aa^ |
| *A. sydowii* PT-2 | 198.3±3.0^Aa^ | 191.1±4.1^ABb^ | 185.6±7.7^Aa^ | 155.8±7.8^Bb^ | 124.6±10.1^Bb^ | 89.2±4.3^Bb^ | 75.5±9.4^Bb^ |
| *A. ustus* PT-6 | 198.3±3.0^Aa^ | 196.9±4.2^Aab^ | 195.3±2.6^Aa^ | 195.3±6.6^Aa^ | 194.0±1.8^Aa^ | 195.2±2.1^Aa^ | 190.8±5.8^Aa^ |
| *A. tamarii* PT-7 | 198.3±3.0^Aa^ | 182.1±2.8^Ac^ | 125.2±14.6^Bb^ | 84.6±8.8^Cc^ | 54.0±9.0^Cc^ | 17.4±7.1^Cc^ | 4.2±3.9^Cc^ |
| Theobromine concentration (mg/L) in the TLM-S with 300 mg/L theobromine. | | | | | | | |
| Isolates | 0 d | 1 d | 2 d | 3 d | 4 d | 5 d | 6 d |
| *A. niger* PT-1 | 299.7±4.2^Aa^ | 299.8±3.4^Aa^ | 298.7±2.8^Aa^ | 295.8±9.8^Aa^ | 297.5±7.3^Aa^ | 289.4±6.1^Aa^ | 296.7±6.7^Aa^ |
| *A. sydowii* PT-2 | 299.7±4.2^Aa^ | 284.9±8.2^Ab^ | 274.2±7.7^Bb^ | 241.3±16.5^Bb^ | 156.3±14.5^Bb^ | 105.7±21.4^Bb^ | 80.5±6.3^Bb^ |
| *A. ustus* PT-6 | 299.7±4.2^Aa^ | 297.5±4.9^Aa^ | 292.6±6.3^Aa^ | 283.6±9.7^Aa^ | 284.2±28.6^Aa^ | 251.8±32.6^Aa^ | 294.8±8.3^Aa^ |
| *A. tamarii* PT-7 | 299.7±4.2^Aa^ | 285.3±6.3^Ab^ | 225.9±7.3^Cc^ | 141.7±14.7^Cc^ | 54.2±7.7^Cc^ | 17.0±7.9^Cc^ | 13.9±2.9^Cc^ |

All data were present by mean value ± SD of three replications. Different lowercase letters in superscript (A, B and C, p<0.01; a, b and c, p < 0.05) in a same row indicate level of theobromine concentration with statically significant difference by Tukey’s multiple comparison test for one-way ANOVA.

**Additional file 1: Table S2** Production of 3-methylxanthine in TLM-S with different substrate concentrations inoculated by *A. sydowii* PT-2 and *A. tamarii* PT-7, respectively.

| 3-Methylxanthine production (mg/L) of *A. sydowii* PT-2 in given TLM-S. | | | | | | |
| --- | --- | --- | --- | --- | --- | --- |
| Concentrations | 1 d | 2 d | 3 d | 4 d | 5 d | 6 d |
| 100 mg/L | 5.58±1.54^Aa^ | 12.87±0.88^Aa^ | 23.79±3.73^Bc^ | 42.00±3.75^Bc^ | 64.73±5.77^Bc^ | 71.84±4.44^Bc^ |
| 200 mg/L | 5.89±1.12^Aa^ | 9.90±2.37^Aa^ | 35.26±5.86^ABb^ | 56.88±10.01^Bb^ | 77.61±6.77^Bb^ | 92.81±2.86^Bb^ |
| 300 mg/L | 8.51±1.56^Aa^ | 14.53±4.16^Aa^ | 46.40±3.54^Aa^ | 111.50±7.20^Aa^ | 155.74±5.81^Aa^ | 177.12±14.06^Aa^ |
| 3-Methylxanthine production (mg/L) of *A. tamarii* PT-7 in given TLM-S. | | | | | | |
| Concentrations | 1 d | 2 d | 3 d | 4 d | 5 d | 6 d |
| 100 mg/L | 8.11±2.65^Aa^ | 17.63±1.90^Aa^ | 28.59±7.15^Bb^ | 42.57±6.76^Bb^ | 44.30±7.48^Ab^ | 49.02±4.21^Ab^ |
| 200 mg/L | 9.74±2.27^Aa^ | 24.82±5.71^Aa^ | 36.72±6.23^ABb^ | 50.13±12.51^ABb^ | 50.87±6.85^Aab^ | 52.34±12.11^Aab^ |
| 300 mg/L | 10.69±1.69^Aa^ | 28.32±8.27^Aa^ | 54.34±8.49^Aa^ | 74.51±9.10^Aa^ | 62.54±10.83^Aa^ | 66.31±5.68^Aa^ |

All data were present by mean value ± SD of three replications. Different lowercase letters in superscript (A and B, p < 0.01; a, b and c, p < 0.05) in a same row indicate level of 3-methylxanthine concentration with statically significant difference by Tukey’s multiple comparison test for one-way ANOVA.

**Additional file 1: Table S3** Related metabolites with 3-methylxanthine degradation detected in the liquid culture of different candidate isolates.

| Metabolite | Candidate isolates | | | |
| --- | --- | --- | --- | --- |
|  | *A. niger* PT-1 | *A. sydowii* PT-2 | *A. ustus* PT-6 | *A. tamarii* PT-7 |
| Theobromine | - | - | - | - |
| 3,7-Dimethyluric acid | - | - | - | - |
| 7-Methylxanthine | - | - | - | - |
| 3-Methyluric acid | - | - | + | + |
| 7-Methyluric acid | - | - | - | - |
| Xanthine | - | + | - | + |
| Uric acid | - | - | - | + |

TLM-S inoculated by candidate isolates were analyzed by HPLC for related metabolites with 3-methylxanthine metabolism, which including theobromine, 3,7-dimethyluric acid, 7-methylxanthine, 3-methyluric acid, 7-methyluric acid, xanthine and uric acid.
